# On the Possibility of Developing Magnetostrictive Fe-Co/Ni Clad Plate with Both Vibration Energy Harvesting and Mass Sensing Elements

**DOI:** 10.3390/ma14164486

**Published:** 2021-08-10

**Authors:** Kotaro Mori, Yinli Wang, Kenichi Katabira, Daiki Neyama, Ryuichi Onodera, Daiki Chiba, Masahito Watanabe, Fumio Narita

**Affiliations:** 1 Department of Mechanical Systems Engineering, College of Engineering, Ibaraki University, Hitachi 316-8511, Japan; kotaro.mori.l@vc.ibaraki.ac.jp; 2 Department of Materials Science, Graduate School of Engineering, Tohoku University, Sendai 980-8579, Japan; wang.yinli.r2@dc.tohoku.ac.jp; 3 Department of Materials Processing, Graduate School of Engineering, Tohoku University, Sendai 980-8579, Japan; kenichi.katabira.s2@dc.tohoku.ac.jp (K.K.); daiki.neyama.r4@dc.tohoku.ac.jp (D.N.); 4 Research and Development Department, Tohoku Steel Co., Ltd., Shibata 989-1393, Japan; r-onodera@tohokusteel.com (R.O.); d-chiba@tohokusteel.com (D.C.); m-watanabe@tohokusteel.com (M.W.); 5 Department of Frontier Sciences for Advanced Environment, Graduate School of Environmental Studies, Tohoku University, Sendai 980-8579, Japan

**Keywords:** electromagneto-mechanics, finite element analysis, energy harvesting test, laminated plates, inverse magnetostrictive effect, output voltage, mass sensors

## Abstract

The severe acute respiratory syndrome coronavirus (SARS-CoV-2) has spread rapidly around the world. In order to prevent the spread of infection, city blockades and immigration restrictions have been introduced in each country, but these measures have a severe serious impact on the economy. This paper examines the possibility of both harvesting vibration energy and detecting mass by using a magnetostrictive alloy. Few efforts have been made to develop new magnetostrictive biosensor materials. Therefore, we propose magnetostrictive Fe-Co/Ni clad steel vibration energy harvesters with mass detection, and we numerically and experimentally discuss the effect of the proof mass weight on the frequency shift and output voltage induced by bending vibration. The results reveal that the frequency and output voltage decrease significantly as the mass increases, indicating that the energy harvesting device is capable of mass detection. In the future, device miniaturization and the possibility of virus detection will be considered.

## 1. Introduction

The Internet of Things, where everything is connected to the Internet, is aimed at introducing innovation to the whole world. Sensors are installed in facilities and equipment, and analog processes are visualized as digital data and then accumulated and analyzed on the ground via the Internet. This allows the possibility of improving quality, efficiency, and productivity, as well as creating new value and services, and the way of doing business in various fields is about to change. Moreover, the number of sensors is expected to increase significantly in the future (number may reach 1 trillion by 2030) [1]. A few microwatts to milliwatts of power is required for driving the sensors and communicating data [2]. However, when using a battery for a trillion sensors, factors such as the environment, resources, and cost must be taken into consideration. For this reason, energy harvesting, which recovers electric power from unused energy (e.g., vibration, heat, light, and radio waves) present in the natural environment, has received increasing attention. The expectation is that this process will be used to recover electric power for sensor driving and data communication.

Piezoelectric ceramics and polymers are expected to exhibit sensing and energy harvesting properties, and many studies have focused on this possibility [3,4,5,6,7,8]. In addition, magnetostrictive alloys are considered promising energy harvesting materials [9,10], and hence the development and characterization of such alloys have attracted attention [11,12]. In recent years, Fe-Ga/Fe-Co laminated materials [13], Fe-Co/piezoelectric laminated materials [14], Tb-Dy-Fe/Fe-Co laminated materials [15], Fe-Co coatings [16], and Fe-Co short wire/polymer composites [17] have been developed for sensor applications. Stress-induced domain wall motion of Co-based microwires [18], inverse magnetostrictive characteristics of Fe-Co wire/Al-Si composites under impact loading [19], and the twisting effect on the energy conversion of Fe-Co wire/Al-Si [20] and Fe-Co wire/polymer [21] composites have also been studied. In addition, an Fe-Co bolt with a sensor function has been developed [22].

In general, vibration energy harvesting devices often use cantilevers, which are capable of harvesting power resulting from bending vibration [23,24]. A large amount of power is obtained by utilizing the resonance phenomenon of the cantilevers. For this reason, many studies have focused on the bending vibration of energy harvesting magnetostrictive cantilevers [25,26,27,28,29].

The expectation is that magnetostrictive materials will serve as biosensors [30]. Guo et al. [31] developed a wireless magnetostrictive biosensor immobilized with E2 glycoprotein to detect the classical swine fever virus (CSFV) E2 antibody. They used Metglas alloy 2826 (Fe_40_Ni_40_P_14_B_6_) and found a linear relationship between the resonance frequency shift and the logarithm of CSFV E2 antibody concentrations ranging from 5 ng mL^−1^ to 10 μg mL^−1^.

Recently, a completely new function has been added to composite materials with the aim of realizing multiple functions at the same time. Research and development of multifunctional composite materials are progressing, and research trends are evolving toward new applications and market expansions of these materials. One strategy for achieving such materials includes simultaneously imparting a magnetostrictive material with both an energy harvesting function and a mass detection function [32].

In the present work, a preliminary study of the Fe-Co/Ni vibration energy harvesting cantilever with mass detection is performed. Finite element analysis was carried out to predict the resonant frequency and output voltage in the Fe-Co/Ni cantilever under bending vibration for various weights of a proof mass. The effects of the mass location and distribution on the frequency shift were examined. The output voltage change due to the mass change was also discussed, and the potential of mass detection was shown by changing the output voltage. The frequency shift and output voltage change due to the mass change were then measured, and the test results were compared with numerical values. This comparison expects a good agreement between the model prediction and test data at the microgram level. The results reveal that the vibration energy harvesting device utilizing the resonance phenomenon can be used as a mass sensor. The findings of this work also provide new insights into the possibility of developing self-powered virus sensors.

## 2. Numerical Procedure

The effects of the location and distribution of a mass on the sensitivity are investigated via finite element analysis. The basic equations for a magnetostrictive material are outlined here. Considering the rectangular Cartesian coordinate system O-*x*_1_*x*_2_*x*_3_, the governing equations are given as follows [33]:

(1) $$\sigma_{ji,j}=\rho u_{i,tt}+cu_{i,t}$$

(2) $$B_{i,i}=0$$

where *σ_ij_*, *B_i_*, and *u_i_* are the stress tensor, magnetic flux density vector, and displacement vector, respectively; *ρ* is the mass density; and *c* is the damping coefficient. A comma followed by an index denotes partial differentiation with respect to the space coordinate *x_i_* or the time *t*, and the summation convention for repeated tensor indices is applied. The constitutive laws are given as follows [34]:

(3) $$\varepsilon_{ij}=s_{ijkl}^{H}\sigma_{kl}+d_{kij}^{m}H_{k}$$

(4) $$B_{i}=d_{ikl}^{m}\sigma_{kl}+\mu_{ik}H_{k}$$

where *ε_ij_* is the strain tensor; *H_i_* is the magnetic field intensity vector; and $s_{ijkl}^{H}$,$\;d_{kij}^{m}$, and *μ_ij_* are the constant magnetic field elastic compliance, piezomagnetic constant, and magnetic permittivity, respectively. The material constants are characterized by the following symmetry conditions:

(5) $$s_{ijkl}^{H}=s_{jikl}^{H}=s_{ijlk}^{H}=s_{klij}^{H},\;d_{kij}^{m}=d_{kji}^{m},\;\mu_{ij}=\mu_{ij}$$

The strain tensor and the displacement vector are related as follows:

(6) $$\varepsilon_{ij}=\frac{1}{2}\left(u_{i,j}+u_{j,i}\right)$$

The magnetic field intensity is expressed as follows:

(7) $$H_{i}=\varphi_{,i}$$

where *φ* is the magnetic potential.

Let us now introduce a compact matrix notation. This notation consists of replacing *ij* or *kl* by *p* or *q*, where *i*, *j*, *k*, and *l* take the values of 1, 2, and 3 and *p* and *q* take the values of 1, 2, 3, 4, 5, and 6 according to Table 1. Thus,

(8) $$s_{ijkl}^{H}=s_{pq}^{H},\;\;d_{ikl}^{m}=d_{ip}^{m}$$

The material properties of Fe-Co and Ni with piezomagnetic effect are transversely isotropic with *x*_3_ being the axis of symmetry [35]. Constitutive Equations (3) and (4) for the magnetostrictive material are given as follows:

(9) $$\left\{\begin{array}{c} \varepsilon_{11}\\ \varepsilon_{22}\\ \varepsilon_{33}\\ 2\varepsilon_{23}\\ 2\varepsilon_{31}\\ 2\varepsilon_{12} \end{array}\right\}=\left[\begin{array}{cccccc} s_{11}^{H} & s_{12}^{H} & s_{13}^{H} & 0 & 0 & 0\\ s_{12}^{H} & s_{11}^{H} & s_{13}^{H} & 0 & 0 & 0\\ s_{13}^{H} & s_{13}^{H} & s_{33}^{H} & 0 & 0 & 0\\ 0 & 0 & 0 & s_{44}^{H} & 0 & 0\\ 0 & 0 & 0 & 0 & s_{44}^{H} & 0\\ 0 & 0 & 0 & 0 & 0 & s_{66}^{H} \end{array}\right]\left\{\begin{array}{c} \sigma_{11}\\ \sigma_{22}\\ \sigma_{33}\\ \sigma_{23}\\ \sigma_{31}\\ \sigma_{12} \end{array}\right\}\;+\left[\begin{array}{ccc} 0 & 0 & d_{31}^{m}\\ 0 & 0 & d_{31}^{m}\\ 0 & 0 & d_{33}^{m}\\ 0 & d_{15}^{m} & 0\\ d_{15}^{m} & 0 & 0\\ 0 & 0 & 0 \end{array}\right]\left\{\begin{array}{c} H_{1}\\ H_{2}\\ H_{3} \end{array}\right\}$$

(10) $$\left\{\begin{array}{c} B_{1}\\ B_{2}\\ B_{3} \end{array}\right\}=\left[\begin{array}{cccccc} 0 & 0 & 0 & 0 & d_{15}^{m} & 0\\ 0 & 0 & 0 & d_{15}^{m} & 0 & 0\\ d_{31}^{m} & d_{31}^{m} & d_{33}^{m} & 0 & 0 & 0 \end{array}\right]\left\{\begin{array}{c} \sigma_{11}\\ \sigma_{22}\\ \sigma_{33}\\ \sigma_{23}\\ \sigma_{31}\\ \sigma_{12} \end{array}\right\}+\left[\begin{array}{ccc} \mu_{11} & 0 & 0\\ 0 & \mu_{11} & 0\\ 0 & 0 & \mu_{33} \end{array}\right]\left\{\begin{array}{c} H_{1}\\ H_{2}\\ H_{3} \end{array}\right\}$$

where

(11) $$\begin{array}{cc} & \sigma_{23}=\sigma_{32}, \end{array}\begin{array}{cc} & \sigma_{31}=\sigma_{13}, \end{array}\begin{array}{cc} & \begin{array}{cc} \sigma_{12}=\sigma_{21} & \end{array} \end{array}$$

(12) $$\begin{array}{cc} & \varepsilon_{23}=\varepsilon_{32}, \end{array}\begin{array}{cc} & \varepsilon_{31}=\varepsilon_{13}, \end{array}\begin{array}{cc} & \begin{array}{cc} \varepsilon_{12}=\varepsilon_{21} & \end{array} \end{array}$$

(13) $$\begin{array}{l} s_{11}^{H}=s_{1111}^{H}=s_{2222}^{H},\begin{array}{cc} & s_{12}^{H}=s_{1122}^{H}, \end{array}\begin{array}{cc} & s_{13}^{H}=s_{1133}^{H}=s_{2233}^{H}, \end{array}\begin{array}{cc} & s_{33}^{H}=s_{3333}^{H}, \end{array}\\ s_{44}^{H}=4s_{2323}^{H}=4s_{3131}^{H},\begin{array}{cc} & s_{66}^{H}=4s_{1212}^{H}=2\left(s_{11}^{H}-s_{12}^{H}\right) \end{array} \end{array}$$

(14) $$d_{15}^{m}=2d_{131}^{m}=2d_{223}^{m},\begin{array}{cc} & d_{31}^{m}=d_{311}^{m}=d_{322}^{m}, \end{array}\begin{array}{cc} & d_{33}^{m}=d_{333}^{m} \end{array}$$

For Fe-Co and Ni used in this study, the constant magnetic field elastic compliances are written in terms of the Young’s modulus *E*, shear modulus *G,* and Poisson’s ratio *ν* as

(15) $$s_{11}^{H}=s_{33}^{H}=\frac{1}{E},\begin{array}{cc} & s_{12}^{H}=s_{13}^{H}=-\frac{\nu }{E}, \end{array}\begin{array}{cc} & s_{44}^{H}=s_{66}^{H}=\frac{1}{G} \end{array}$$

The relationship between engineering constants is given by *G* = *E*/{2(1 + *ν*)}.

Many theoretical studies on magnetostrictive biosensors have been performed [32]. Similarly, for simple shapes, solutions for the resonance frequency and the frequency change due to a mass change have been discussed. The fundamental resonant frequency *f*_0_ of a thin plate with length *l*, width *w* and thickness *h* (*h*, *w*≪*l*) vibrating in the longitudinal mode is given as follows [36]:

(16) $$f_{0}=\frac{1}{2l}\sqrt{\frac{E}{\rho (1-\nu^{2})}}$$

If the mass increase is small compared with the initial mass *ρlwh* of the plate, then the mass change, in accordance with the measured resonance frequency shift, is determined from the following equation [37]:

(17) $$\Delta m=-2\rho lwh\frac{\Delta f}{f_{0}}$$

Equation (17) indicates that the resonance frequency decreases linearly with increasing mass on the plate surface. However, evaluating the frequency change from Equation (17) is impossible for the cantilever under bending vibration, and hence, we used the finite element analysis software ANSYS to perform the evaluation.

**Figure 1:**
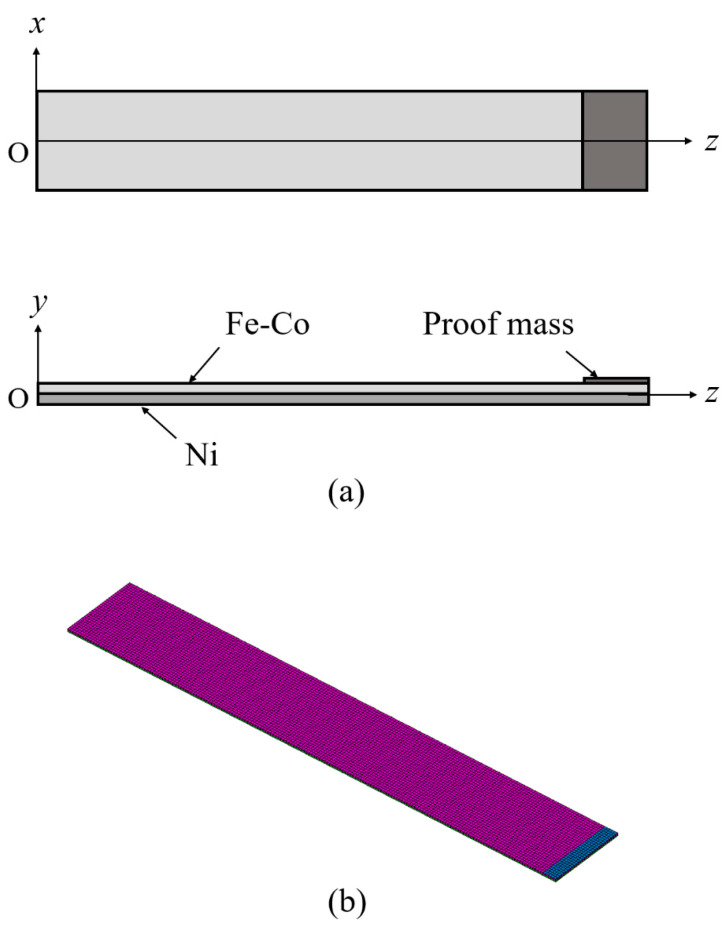
Finite element model of the Fe-Co/Ni clad plate. (**a**) geometry and (**b**) finite element mesh.

A magnetostrictive clad plate cantilever with the Fe-Co layer (length *l*, width *w*, and thickness *h*/2) perfectly bonded to the top surface of the Ni layer (length *l*, width *w*, and thickness *h*/2) is shown in Figure 1. The proof mass layer (length *L*, width *W* = *w*, and hight *H*) with weight *m* was attached to the cantilever. Dimensions *w*, *h*, and *l* were measured along the *x*_1_ = *x*, *x*_2_ = *y*, and *x*_3_ = *z* axes, respectively. The origin of the coordinate system is located at the left-side center of the clad plate. The plate was clamped with *z* = 0 denoting the fixed center. The *z*-direction was the easy axis of magnetization for both the Fe-Co and Ni layers. The imposed base excitation was given by the displacement *u_y_*_0_exp(*iωt*) of the clamped end, where *u_y_*_0_ is the amplitude of the applied displacement and *ω* = 2π*f* is the angular frequency. Furthermore, the mass change induced changes in the magnetic flux density and frequency of the clad plate were calculated via finite element analysis. Moreover, through Faraday’s law, the output voltage was obtained from the magnetic flux density change as follows:

(18) $$V_{\mathrm{out}}=-Nwh\frac{dB_{z}}{dt}$$

where *N* is the number of turns of a coil. Table 2 and Table 3 list the material properties of the Fe-Co and Ni layers.

## 3. Experiment

When a clad plate with Fe-Co and Ni bonded via thermal diffusion is subjected to bending vibration, the vibration power output obtained is larger than that realized from a traditional Fe-Co plate [26]. A cantilever beam of Fe-Co/Ni clad plate thinner than those used in previous studies [26,38] was fabricated in the present study. The length, width, and thickness were 50 mm, *w* = 6.2 mm, and *h* = 0.2 mm, respectively.

**Figure 2:**
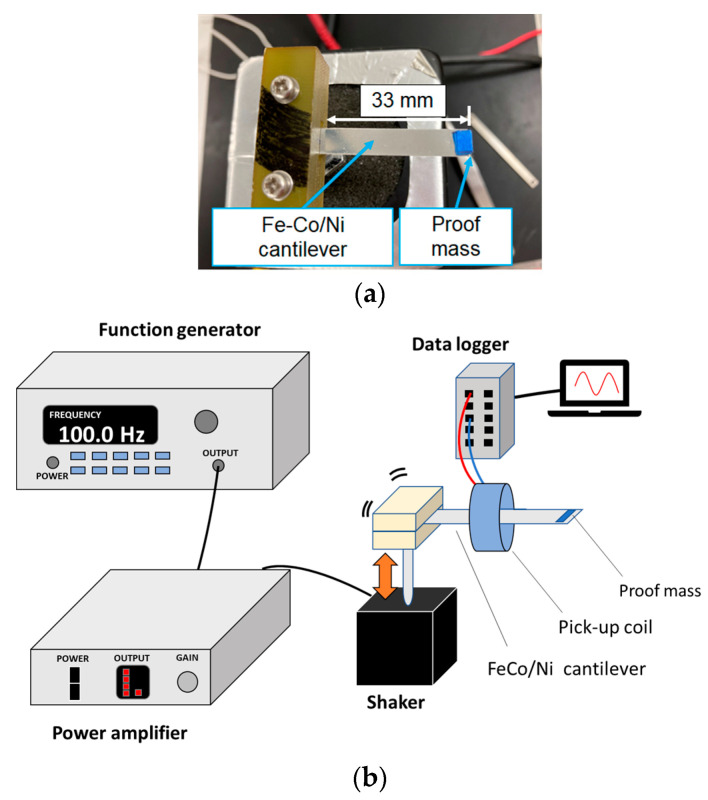
Schematic of (**a**) specimen and (**b**) experimental setup.

To measure the output voltage *V*_out_ of the Fe-Co/Ni clad plate, a vibration power generation test was executed using a vibrator. The clad plate cantilever with a proof mass of *m* = 1, 2, 4, 7, or 10 mg was mounted on the vibration shaker (ET-132, Labworks Inc., Costa Mesa, CA, USA), and the imposed displacement vibration was applied with the shaker. The proof mass was a masking tape made of paper. A cantilever without the mass was also used. The free length of the cantilever was *l* = 33 mm. The peak-to-peak output voltage *V*_pp_ = 2*V*_out_ associated with various values of the frequency *f* was recorded using a data logger (NR-500 series, Keyence Corporation, Osaka, Japan). The number of turns of the coil, resistance of the coil, and the sampling period were ~4200, 7.47 kΩ, and 100 μs, respectively. Figure 2 shows the specimen and experimental setup.

## 4. Results and Discussion

**Figure 3:**
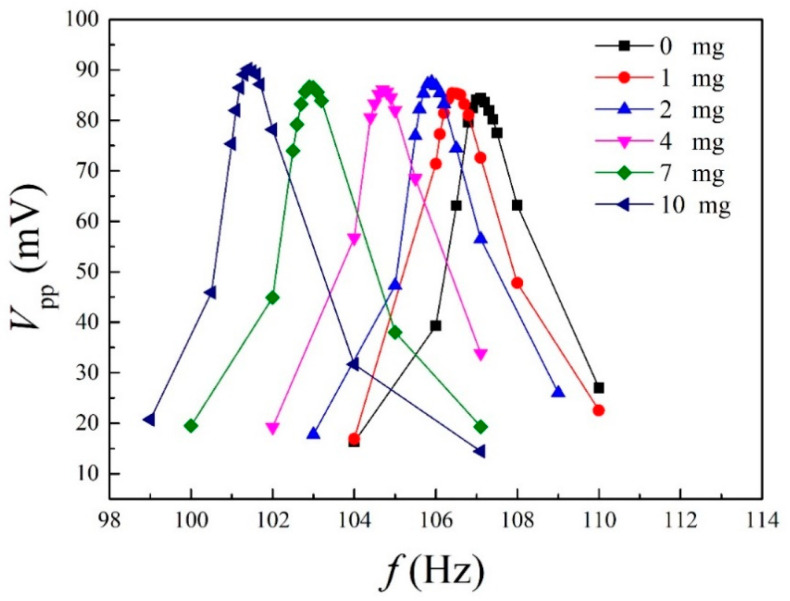
Measured output voltage versus frequency.

**Figure 4:**
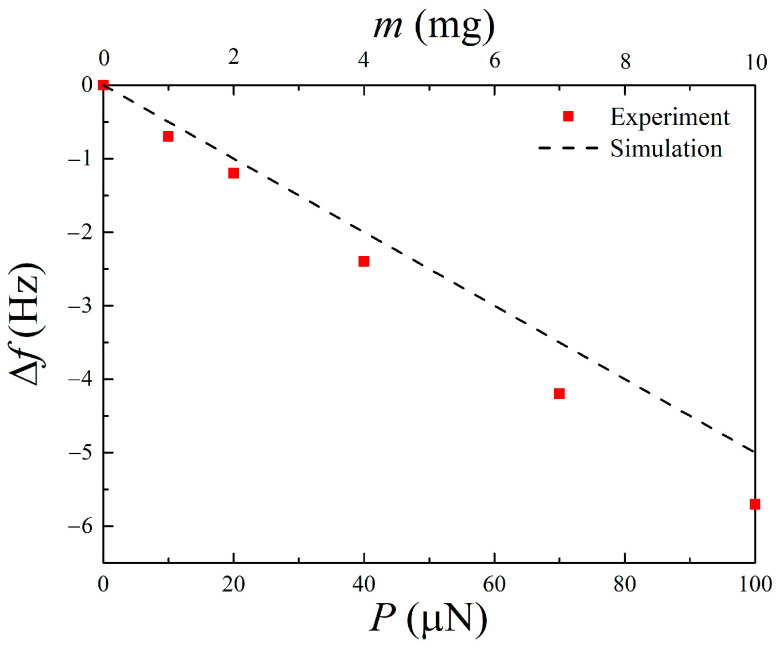
Measured and calculated frequency shift versus load or weight.

Figure 3 shows the measured peak-to-peak output voltage *V*_pp_ versus frequency *f* for the Fe-Co/Ni clad plates with a proof mass of *m* = 0, 1, 2, 4, 7, and 10 mg. The resonance frequency of the Fe-Co/Ni clad plate without the proof mass was ~107 Hz. It was expected that for the first mode, the maximum output voltage generated from the Fe-Co/Ni clad plate without the proof mass would be ~85 mV. As the weight of the mass increases, the frequency decreases, and the maximum output voltage increases slightly. Table 4 presents a comparison of the output voltage density of the Fe-Co/Ni clad plate with the magnetostrictive harvesters [21,25,27,29,39,40]. Although the output voltage density depends on the bias magnetic field, excitation conditions, number of coil turns, etc., the performance of the Fe-Co/Ni clad plate is almost comparable to those of other magnetostrictive harvesters. Figure 4 shows the measured and calculated frequency shift Δ*f* (due to the proof mass) as a function of the load *P* or weight *m* of the mass. The damping ratios *ζ* = *c*/*c*_c_ (*c*_c_: critical damping coefficient) of the Fe-Co/Ni clad plate with a proof mass of *m* = 0, 1, 2, 4, 7, and 10 mg were obtained by the half power method and were 0.0084, 0.0094, 0.0085, 0.0091, 0.0087, and 0.0094. The length *L* and height *H* of the mass were 1 mm and 0.1 mm, respectively. The frequency shift decreases linearly with increasing load or weight. In addition, the mass can be detected from changes in the resonance frequency of this clad steel plate.

**Figure 5:**
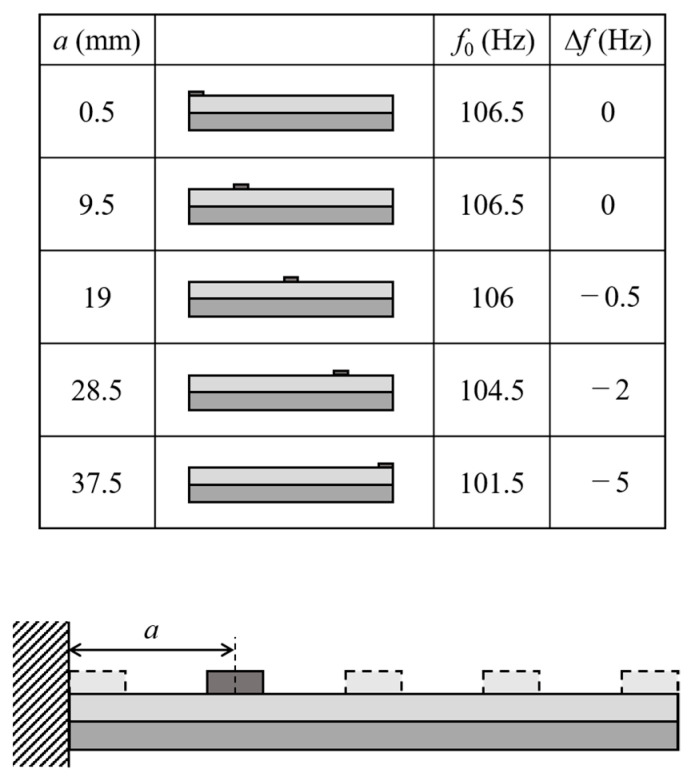
Calculated resonance frequency and frequency shift for various values of the distance between the weight and the clamped end.

**Figure 6:**
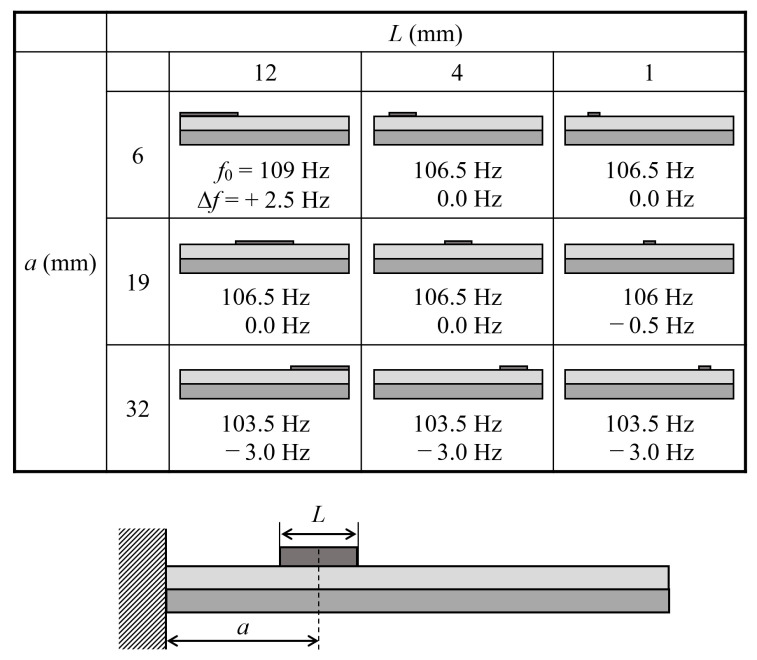
Calculated resonance frequency and frequency shift for various values of the distance between the proof mass and the clamped end and the length of the mass.

A recent study [41] has shown that optimizing the location and distribution of the load provides the highest sensitivity. Figure 5 shows the calculated resonance frequency *f*_0_ of the Fe-Co/Ni clad plates where the distance between the center of the proof mass and the fixed end is *a* = 0.5, 9.5, 19, 28.5, and 37.5 mm. The length *L* and height *H* of the mass were 1 mm and 0.1 mm, respectively. The calculated frequency shifts Δ*f* due to the mass of weight *m* = 10 mg are shown in the figure. The resonance frequency increases (as expected) with decreasing distance of the proof mass from the fixed end, whereas the frequency shift decreases. Figure 6 shows the calculated resonance frequency *f*_0_ and frequency shift Δ*f* due to the mass with weight *m* = 10 mg of the Fe-Co/Ni clad plates. The *f*_0_ and Δ*f* values were calculated for various values of *a* (the distance between the proof mass and the fixed end) and *L* (the length; *L* = 12, 4, and 1 mm) of the proof mass. When the proof mass is near the fixed end, the frequency increases with the mass as the size of the mass increases even if the weight is the same. The frequency changes only slightly when the proof mass is in the center of the cantilever. When the mass is near the free end, the frequency is reduced by the mass regardless of the mass size.

**Figure 7:**
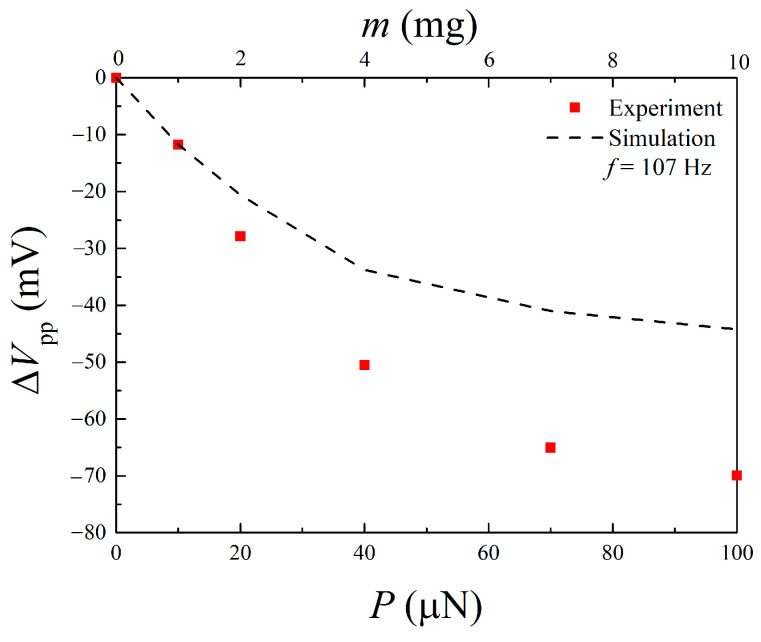
Measured and calculated output voltage change versus load or weight.

Figure 7 shows the change in the peak-to-peak output voltage Δ*V*_pp_ versus the load *P* or weight *m* obtained from the experiment and finite element analysis. The voltage decreases by ~12 mV at a weight of *m =* 1 mg. The results shown in this figure suggest that the output voltage decreases linearly due to the mass change at the microgram level.

The results obtained indicate that the Fe-Co/Ni clad plate with energy harvesting capability can detect the weight of the proof mass. In particular, a weight of ~1 mg can be detected from a change in the output voltage of 12 mV. The output voltage of the magnetostrictive material increases proportionally to the number of coil turns [42]. Therefore, the detected weight decreases with the increasing number of coil turns.

**Figure 8:**
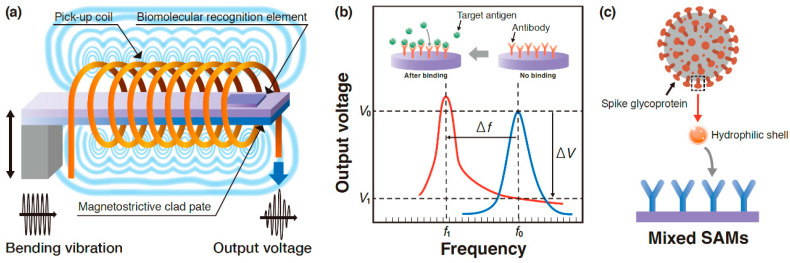
Schematic of (**a**) magnetostrictive harvester, (**b**) voltage to frequency during detection, and (**c**) bio-interfacial interaction of spike glycoprotein with mixed SAMs.

The principle of magnetostrictive biosensors is as follows [43]: Due to the magnetostrictive effect, an alternating current (AC) magnetic field applied to the magnetostrictive laminated plate would result in a bending vibration and generate a physical resonance. Consider the surface of the plate covered with a biomolecular recognition element such as an antibody. The fundamental resonance frequency *f*_0_ of the plate without antigen binding decreases to *f*_1_ owing to the fact that antigen binds to the antibody immobilized on the sensor surface. This shift of the resonance frequency can be monitored by using a pickup coil. Our Fe-Co/Ni clad plate serves as the bending vibration energy harvesting device, and without antigen adsorption, the harvested power will be able to supply as a source of transmitting power in order to forward information from the sensor. Furthermore, this plate for energy harvesting is subjected to bending vibration and serves as the biosensor without the need for an AC magnetic field (see Figure 8a). In addition, our biosensor may be able to detect the mass from the reduced output voltage Δ*V* = *V*_0_ − *V*_1_, which will significantly reduce the detection time (Figure 8b). The purpose of this study is to confirm the principles of vibration energy harvesting and mass detection. Hence, the “mass” of the mass sensing element is not specifically identified. In future research, experiments will be conducted on the mass of the virus. The comparison between two types of traditional biosensors [32,44] and our future biosensor is given in Table 5.

According to a recent study, quartz crystal sensors exhibiting the piezoelectric effect can be modified with mixed self-assembled monolayers (SAMs) and can be explored for the label-free detection of severe acute respiratory syndrome coronavirus 2 (SARS-CoV-2) [45]. The role played by engineered surface chemistry of personal protective equipment in avoiding the spread of the novel SARS-CoV-2 virus in hospitals was also highlighted [46]. These engineered surfaces could be reused after desorption. Future work could consider the implementation of these mixed SAMs on the Fe-Co/Ni clad plates (Figure 8c).

## 5. Conclusions

In this study, the effect of mass on the bending vibration power generation behavior of a magnetostrictive clad plate was investigated, and the biosensor was reasonably designed based on this principle. Besides, the feasibility was verified by both experiment and numerical simulation method. The voltage output via this vibration decreases with the increasing weight of the mass, owing to the fact that the resonance frequency is shifted by the mass. Through various results, it was confirmed that the detection of the microgram level can be achieved. What is more, the optimization of the biosensor was also conducted to reach the best performance in, for example, detecting the position and size of an antibody. Compared with the traditional detection method, the benefit of the detection from changes of the output voltage instead of the frequency shift is that it does not require fast Fourier transform or discrete Fourier transform analysis and will shorten the detection time. Therefore, real-time detection can be realized, and it is easier to read the results. Since AC magnetic field is not necessary for this detection system, the device can be made wirelessly. Moreover, the materials used in this study are relatively cheap, and the manufacturing process is simple, which is very helpful for wide application. However, the sensitivity of this detection system is not sufficient due to the nanogram scale of viruses. Further research is needed and is currently underway to confirm this possibility.

## Figures and Tables

**Table 1:** Extended tensor notation and compressed matrix notation.

*ij* or *kl*	*p* or *q*
11	1
22	2
33	3
23 or 32	4
31 or 13	5
12 or 21	6

**Table 2:** Elastic compliance and mass density of the Fe-Co and Ni layer.

Material	Elastic Compliance * (×10^−12^ m^2^/N)						Mass Density (kg/m^3^)
	$s_{11}$	$s_{33}$	$s_{44}$	$s_{66}$	$s_{12}$	$s_{13}$	
Fe-Co	5.5	5.5	14.3	14.3	−1.65	−1.65	8400
Ni	5.0	5.0	13.1	13.1	−1.55	−1.55	8900

* Superscript H on the elastic compliance has been dropped.

**Table 3:** Piezomagnetic constant and magnetic permittivity of the Fe-Co and Ni layer.

Material	Piezomagnetic Constant * (×10^−12^ m^2^/A)			Magnetic Permittivity (×10^−6^ H/m)	
	$d_{31}$	$d_{33}$	$d_{15}$	*μ*_11_	*μ*_33_
Fe-Co	−60.3	125	318	1.26	1.26
Ni	35.5	−73.6	−187.2	2.51	2.51

* Superscript m on the piezomagnetic constant has been dropped.

**Table 4:** Comparison of the output voltage densities of magnetostrictive cantilevers under bending vibration.

Material	Output Voltage Density (V/cm^3^)	Bias Magnetic Field	Condition	Reference
Fe-Co/Ni clad plate	2.1	0 mT	Frequency of 107 Hz	This work
Fe-Co wire/epoxy Permendur plate	15 0.0044	0 mT A permanent magnet	Frequency of 158 Hz Frequency of 64 Hz	[21] [27]
Fe-Ga plate	0.25	0 mT	Frequency of 75 Hz 5 g acceleration	[29]
	2.3	8 permanent magnets	Frequency of 75 Hz	[29]
Fe-Ga plate Fe-Ga/Cu laminate	0.34 4.0	6.25 mT 8 permanent magnets	2 g acceleration Frequency of 180 Hz Frequency of 25 Hz 1 g acceleration	[39] [40]
Fe-Ga/piezoelectric laminate	0.6	0.35 mT	Frequency of 105 Hz 0.1 g acceleration	[25]

**Table 5:** Comparison of different biosensors.

	Traditional Piezoelectric	Traditional Magnetostrictive	Present Magnetostrictive
Structure	Complicated	Simpler	Simpler
Fabrication	Difficult	Easy	Easy
Actuating	Electrical	Magnetic	Ambient vibration
Sensing	Electrical	Magnetic	Magnetic
Measured value	Frequency shift	Frequency shift	Output voltage
Advantage	Compact configuration	Simple configuration	Simple configuration
	-	High flexibility	High flexibility
	-	Wireless	Wireless
	-	-	Rapid
Disadvantage/future work	Brittleness	Pick-up coil	Pick-up coil
	Charge leakage	Nonlinear effect	Need to improve sensitivity
		Eddy current	

## Data Availability

The data presented in this study are available on request from the corresponding author.

## References

[1] Bryzek J. Proceedings of the TSensors Summit.

[2] Narita F., Fox M. (2018). A review on piezoelectric, magnetostrictive, and magnetoelectric materials and device technologies for energy harvesting applications. Adv. Eng. Mater..

[3] Wang Z., Narita F. (2019). Fabrication of potassium sodium niobate nano-particle/polymer composites with piezoelectric stability and their application to unsteady wind energy harvesters. J. Appl. Phys..

[4] Hara Y., Yamamoto Y., Makihara K. (2020). Self-sensing state estimation of switch-controlled energy harvesters. J. Intell. Mater. Syst. Struct..

[5] Khazaee M., Rezaniakolaie A., Rosendahl L. (2020). A broadband macro-fiber-composite piezoelectric energy harvester for higher energy conversion from practical wideband vibrations. Nano Energy.

[6] Wang Z., Kurita H., Nagaoka H., Narita F. (2020). Potassium sodium niobate lead-free piezoelectric nanocomposite generators based on carbon-fiber-reinforced polymer electrodes for energy-harvesting structures. Compos. Sci. Technol..

[7] Wang Y., Yanaseko T., Kurita H., Sato H., Asanuma H., Narita F. (2020). Electromechanical response and residual thermal stress of metal-core piezoelectric fiber/Al matrix composites. Sensors.

[8] Hara Y., Zhou M., Li A., Otsuka K., Makihara K. (2021). Piezoelectric energy enhancement strategy for active fuzzy harvester with time-varying and intermittent switching. Smart Mater. Struct..

[9] Atulasimha J., Flatau A.B. (2011). A review of magnetostrictive iron–gallium alloys. Smart Mater. Struct..

[10] Deng Z., Dapino M.J. (2017). Review of magnetostrictive vibration energy harvesters. Smart Mater. Struct..

[11] Nakajima T., Takeuchi T., Yuito I., Kato K., Saito M., Abe K., Sasaki T., Sekiguchi T., Yamaura S. (2014). Effect of annealing on magnetostrictive properties of Fe–Co alloy thin films. Mater. Trans..

[12] Yamaura S., Nakajima T., Satoh T., Ebata T., Furuya Y. (2015). Magnetostriction of heavily deformed Fe–Co binary alloys prepared by forging and cold rolling. Mater. Sci. Eng. B.

[13] Liu L., Zhan Q., Yang H., Li H., Zhang S., Liu Y., Wang B., Tan X., Li R.-W. (2016). Magnetostrictive GMR spin valves with composite FeGa/FeCo free layers. AIP Adv..

[14] Bennett S.P., Baldwin J.W., Staruch M., Matis B.R., LaComb J., Jvan’tErve O.M., Bussmann K., Metzler M., Gottron N., Zappone W. (2017). Magnetic field response of doubly clamped magnetoelectric microelectromechanical AlN-FeCo resonators. Appl. Phys. Lett..

[15] Zhu L., Li K., Luo Y., Yu D., Wang Z., Wu G., Xie J., Tang Z. (2019). Magnetostrictive properties and detection efficiency of TbDyFe/FeCo composite materials for nondestructive testing. J. Rare Earths.

[16] Wang W., Jia Y., Xue X., Liang Y., Du Z. (2018). Magnetostrictive effect in micro-dotted FeCo film coated surface acoustic wave devices. Smart Mater. Struct..

[17] Wang Z., Mori K., Nakajima K., Narita F. (2020). Fabrication, modeling and characterization of magnetostrictive short fiber composites. Materials.

[18] Bhatti S., Ma C., Liu X., Piramanayagam S.N. (2019). Stress-induced domain wall motion in FeCo-based magnetic microwires for realization of energy harvesting. Adv. Electron. Mater..

[19] Seino M., Jiang L., Yang Z., Katabira K., Satake T., Narita F., Murasawa G. (2020). Impact energy harvesting by Fe-Co fiber reinforced Al-Si matrix composite. Materialia.

[20] Yang Z., Wang Z., Seino M., Kumaoka D., Murasawa G., Narita F. (2020). Twisting and reverse magnetic field effects on energy conversion of magnetostrictive wire metal matrix composites. Phys. Status Solidi RRL.

[21] Yang Z., Wang Z., Nakajima K., Neyama D., Narita F. (2021). Structural design and performance evaluation of FeCo/epoxy magnetostrictive composites. Compos. Sci. Technol..

[22] Mori K., Horibe T., Maejima K. (2020). Evaluation of the axial force in an FeCo bolt using the inverse magnetostrictive effect. Measurement.

[23] Ueno T., Yamada S. (2011). Performance of energy harvester using iron–gallium alloy in free vibration. IEEE Trans. Magn..

[24] Mori K., Horibe T., Ishikawa S., Shindo Y., Narita F. (2015). Characteristics of vibration energy harvesting using giant magnetostrictive cantilevers with resonant tuning. Smart Mater. Struct..

[25] Xu X., Zhang C., Han Q., Chu F. (2018). Hybrid energy harvesting from mechanical vibrations and magnetic field. Appl. Phys. Lett..

[26] Yang Z., Nakajima K., Onodera R., Tayama T., Chiba D., Narita F. (2018). Magnetostrictive clad steel plates for high-performance vibration energy harvesting. Appl. Phys. Lett..

[27] Ghodsi M., Ziaiefar H., Mohammadzaheri M., Al-Yahmedi A. (2019). Modeling and characterization of permendur cantilever beam for energy harvesting. Energy.

[28] Patra S. (2020). Design and development of magnetostrictive low power DC generator and vibration sensor. IEEE Sens. J..

[29] Liu H., Cong C., Cao C., Zhao Q. (2020). Analysis of the key factors affecting the capability and optimization for magnetostrictive iron-gallium alloy ambient vibration harvesters. Sensors.

[30] Saberkari H., Ghavifekr H.B., Shamsi M. (2015). Comprehensive performance study of magneto cantilevers as a candidate model for biological sensors used in lab-on-a-chip applications. J. Med. Signals Sens..

[31] Guo X., Sang S., Guo J., Jian A., Duan Q., Ji J., Zhang Q., Zhang W. (2017). A magnetoelastic biosensor based on E2 glycoprotein for wireless detection of classical swine fever virus E2 antibody. Sci. Rep..

[32] Narita F., Wang Z., Kurita H., Li Z., Shi Y., Jia Y., Soutis C. (2021). A review of piezoelectric and magnetostrictive biosensor materials for detection of COVID-19 and other viruses. Adv. Mater..

[33] Alshits V.I., Darinskii A.N., Lothe J. (1992). On the existence of surface waves in half-infinite anisotropic elastic media with piezoelectric and piezomagnetic properties. Wave Motion.

[34] Engdahl G. (2000). Handbook of Giant Magnetostrictive Materia1-9ls.

[35] Lee J., Boyd J.G., Lagoudas D.C. (2005). Effective properties of three-phase electro-magneto-elastic composites. Int. J. Eng. Sci..

[36] Liang C., Morshed S., Prorok B.C. (2007). Correction for longitudinal mode vibration in thin slender beams. Appl. Phys. Lett..

[37] Grimes C.A., Ong K.G., Loiselle K., Stoyanov P.G., Kouzoudis D., Liu Y., Tong C., Tefiku F. (1999). Magnetoelastic sensors for remote query environmental monitoring. Smart Mater. Struct..

[38] Yang Z., Onodera R., Tayama T., Watanabe M., Narita F. (2019). Magnetomechanical design and power generation of magnetostrictive clad plate cantilever. Appl. Phys. Lett..

[39] Liu H., Lim C.W., Gao S., Zhao J. (2019). Effects analysis of bias and excitation conditions on power output of an environmental vibration energy harvesting device using Fe-Ga slice. Mechatronics.

[40] Liu H., Li W., Sun X., Cong C., Cao C., Zhao Q. (2021). Enhanced the capability of magnetostrictive ambient vibration harvester through structural configuration, pre-magnetization condition and elastic magnifier. J. Sound Vib..

[41] Wang Y., Shi Y., Narita F. (2021). Design and finite element simulation of metal-core piezoelectric fiber/epoxy matrix composites for virus detection. Sens. Actuators A Phys..

[42] Narita F., Katabira K. (2017). Stress-rate dependent output voltage for Fe_29_Co_71_ magnetostrictive fiber/polymer composites: Fabrication, experimental observation and theoretical prediction. Mater. Trans..

[43] Guntupalli R., Hu J., Lakshmanan R.S., Huang T.S., Barbaree J.M., Chin B.A. (2007). A magnetoelastic resonance biosensor immobilized with polyclonal antibody for the detection of Salmonella typhimurium. Biosens. Bioelectron..

[44] Zhang K., Zhang L., Fu L., Li S., Chen H., Cheng Z.-Y. (2013). Magnetostrictive resonators as sensors and actuators. Sens. Actuators A.

[45] Pandey L.M. (2020). Design of engineered surfaces for prospective detection of SARS-CoV-2 using quartz crystal microbalance-based techniques. Expert Rev. Proteom..

[46] Pandey L.M. (2020). Surface engineering of personal protective equipments (PPEs) to prevent the contagious infections of SARS-CoV-2. Surf. Eng..

